# Low-Frequency Self-Powered Footstep Sensor Based on ZnO Nanowires on Paper Substrate

**DOI:** 10.1186/s11671-016-1373-1

**Published:** 2016-03-22

**Authors:** E. S. Nour, A. Bondarevs, P. Huss, M. Sandberg, S. Gong, M. Willander, O. Nur

**Affiliations:** Department of Science and Technology (ITN), Linköping University, Campus Norrkoping, SE-60 174 Norrköping, Sweden; Printed Electronics, Acreo AB, P.O. Box 787, 60117 Norrköping, Sweden

**Keywords:** ZnO, Hydrothermal growth, Piezoelectric nanowire, Nanogenerator, Energy harvesting, Wireless data transmission

## Abstract

In this work, we design and fabricate a wireless system with the main operating device based on zinc oxide (ZnO) nanowires. The main operating device is based on piezoelectric nanogenerator (NG) achieved using ZnO nanowires grown hydrothermally on paper substrate. The fabricated NG is capable of harvesting ambient mechanical energy from various kinds of human motion, e.g., footsteps. The harvested electric output has been used to serve as a self-powered pressure sensor. Without any storage device, the signal from a single footstep has successfully triggered a wireless sensor node circuit. This study demonstrates the feasibility of using ZnO nanowire piezoelectric NG as a low-frequency self-powered sensor, with potential applications in wireless sensor networks.

## Background

The information technology is a major driving force for the modern world’s development. The collection and exchange of information rely on various types of sensor networks with different functionalities. The construction of large-scale sensor networks and systems can help realize the concept of “Internet of Things,” which correlates objects and devices to large data bases and networks. By replacing the traditional and discrete sensors with a large number of independent and mobile sensors distributed in the field, a statistical analysis of the signals collected through the Internet can hence provide precise and reliable information. In many cases, the sensor nodes are distributed across a wide range of area or embedded/implanted in closed locations. If these sensors can operate through a wireless network, it would imply a huge potential for many applications, e.g., implantable biosensors, patient monitoring, environmental and structure monitoring, and even for national security applications [1]. For these systems, it is very important for the sensor nodes to have the capability of operating independently, sustainably, and with maintenance-free options. However, under the context of the current technology, most of the sensors need power sources for driving their operation. These power sources cannot simply be provided by batteries, for two reasons: (i) the number of sensors to be involved in the sensor network will be huge and their locations could be difficult to track and (ii) the periodic replacement of batteries will create a huge amount of materials that are environmentally unfriendly and potentially hazardous to human health. Therefore, realizing self-powered operation for the sensor nodes in wireless sensor networks is critically important, which is gradually becoming a major research direction in this area now and probably in the near future [2, 3]. The most feasible way to achieve self-powered operation is to harvest energy from ambient sources to drive the sensor node itself [4]. The energy-harvesting device can directly serve as a sustainable power source for the sensor node or at least to be used together with a battery to replenish its energy consumption. Besides, if the sensor can generate electric signal as a response to a low-frequency trigger or change in the environment, it can operate without an external power source. With this strategy, the sensor system can be simplified and the total energy consumption can largely be reduced. Thus, developing self-powered active sensors can largely facilitate the wide range of applications for wireless sensor networks. In 2006, Z. L. Wang’s group has developed a new nanogenerator (NG) technology for converting mechanical energy into electricity [5, 6]. Besides the outstanding capability of serving as a sustainable power source for electronic devices, the produced electric signals from NGs can also be utilized for directly sensing an external mechanical action without a power source. The first type of nanogenerators is based on the piezoelectric effect of semiconductor nanowires (e.g., zinc oxide (ZnO), gallium nitride (GaN)) [5, 7–10].

One important feature of an efficient piezoelectric harvesting system is that it should produce high output at a low operating frequency. Zinc oxide is emerging as an attractive material for harvesting mechanical power, as it is one of the best piezoelectric materials. In addition, ZnO is a semiconductor that possesses a direct band gap of 3.34 eV and a relatively large exciton binding energy of 60 meV. Moreover, ZnO has a high stable non-centro-symmetric hexagonal wurtzite structure leading to a relatively large piezoelectric coefficient, high modulus of elasticity and high piezoelectric tensor. In fact, ZnO has for some years attracted the research community’s attention due to these excellent properties, combined with the fact that in the nanostructure form, many advantages can be utilized. ZnO possesses the richest family of different morphologies, possible to obtain using a variety of physical and chemical synthesis techniques. On the one hand, a number of research articles have been published on the piezoelectric properties of ZnO nanostructures, such as nanorods (NRs), nanowires (NWs), nanoneedles (NNs), nanobelts (NBs), and nanoflowers (NFs) [11]. On the other hand, poly vinylidene fluoride-trifluoroethylene (PVDF-TrFE) and other copolymers have shown to provide the best electroactive performance in displaying piezo-, pyro-, and ferroelectricity characteristics. These properties originate from the strong molecular dipoles within the polymer chains [12] and can result in an enhanced amount of output.

Owing to an increasing demand for green electronics, developments of devices that can generate power have attracted great attention in recent years [13]. Paper, formed by multiple layers of cellulose fibers, has been recognized as one of the most environmentally friendly material. In addition, paper possesses the advantages of flexibility, light weight, low cost, and recyclability; once an electronic device is fabricated with or using paper, a broad technology impact will be created in the field of renewable and sustainable energy [14, 15]. Therefore, several attempts have been made in innovating paper-based electronics for energy storage and conversion applications, such as Li-ion batteries [16], self-powered super-capacitors [17, 18], solar cells [19, 20], and nanogenerators [21, 22].

In this paper, ZnO NWs combined with PVDF-TrFE have been used as the main piezoelectric materials. We demonstrate an application of PVDF-TrFE pasted on the top of the ZnO NWs with silver electrodes and used as an acoustic actuator and highly efficient NG. The resulting silver/ZnO NWs + PVDF-TrFE/silver NG exhibited good durability and sensitivity. By applying a single footstep on the NG, the harvested energy has been used to efficiently trigger a wireless sensor network. The adopted process is compatible with conventional batch fabrication steps and hence can be used for mass production. The grown/deposited material was subjected to complementary structural characterization. A single and multiple NGs have been utilized as wireless sensors providing a stable and strong signal transfer process.

## Method

### Growth Method

The paper substrates used in our experiments were cut from a large piece of common packing paper with high flexibility (Invercote G from Holmen AB, Sweden). After being cleaned ultrasonically in acetone and ethanol, a 10/50-nm layer of chrome/silver was evaporated on the paper substrate to act as a contact. Then, this paper substrate coated with silver was cleaned by acetone, deionized water, and isopropanol, respectively. Further, the substrate preparation technique developed by Green et al. [23] was used to improve the quality of the grown nanorods. The growth of ZnO NWs was achieved using equimolar concentrations (0.075 M) of both hexamethylenetetramine (HMT) and zinc nitrate hexahydrate. Then, the prepared solution was poured in a beaker, and the pre-treated substrates were immersed in the solution with the growth side facing downward. Afterwards, the beaker was sealed and heated in a laboratory oven at 90 °C for 6 h, and then, it was allowed to cool down to room temperature. After the growth process, to remove the residual salts, the samples were rinsed with deionized water and dried with flowing nitrogen.

### Device Fabrication and Measurements

**Fig. 1:**
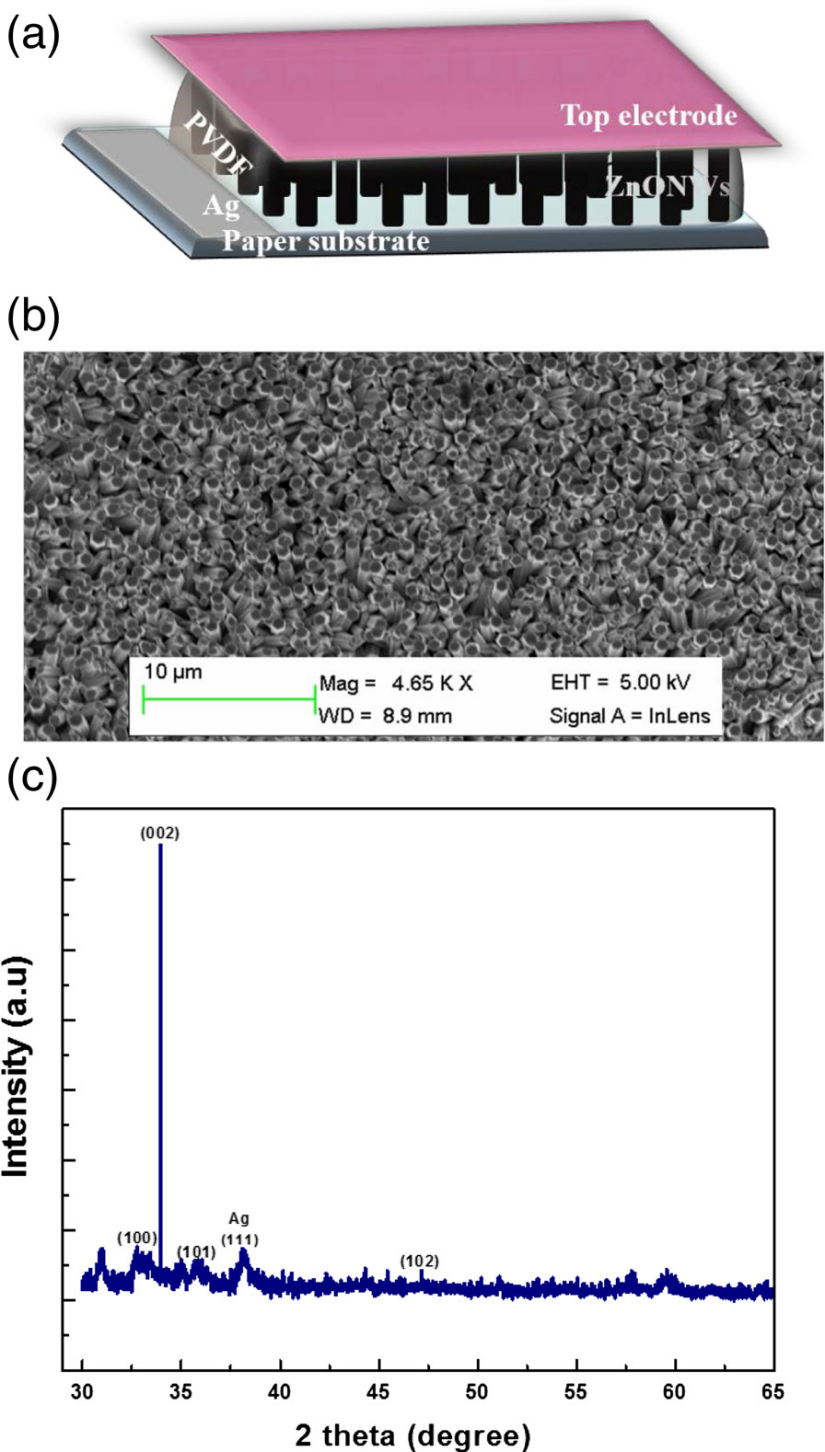
**a** Schematic diagram showing the NGs device. **b** SEM image of the ZnO NWs grown on the paper substrate. **c** XRD patterns of the ZnO nanowires grown on the paper substrate

For the NG device fabrication, a layer of PVDF-TrFE of a few micrometers was pasted on the top of the as grown ZnO NWs on the paper substrate. Then, a silver coated plastic has been used as a top contact for the measurements. The sample was left for 2 to 3 days, and then, the measurements were performed. The edges of the two pieces of the samples were connected by electric wires to an oscilloscope, in order to measure the piezoelectric harvested potential of the fabricated NGs. A schematic diagram depicts the structure of the fabricated NG on the flexible paper substrate is shown in Fig. 1a.

## Results and Discussion

### Surface Morphology and Structure Characterization

The structural characterization of the grown nanostructures was performed using scanning electron microscopy (SEM) and powder X-ray diffraction (XRD). Figure 1b shows a low magnification SEM micrograph of the ZnO NWs grown on the paper substrate. As clearly seen, dense, homogeneous, and well-aligned ZnO NWs arrays were achieved. The X-ray diffraction pattern from this sample is shown in Fig. 1c. Three diffraction peaks of the {(100), (002), and (101)} are well consistent with the hexagonal peaks of diffraction phase of ZnO and in agreement with the JCPDS Card No. [36-1451] file. The (002) reflection peak is intense and sharper in nature, as compared to other peaks, indicating a preferential c-axis growth orientation of the NWs. In addition to that, a (111) Ag diffraction peak was observed from the substrate.

### Performance of the NG

**Fig. 2:**
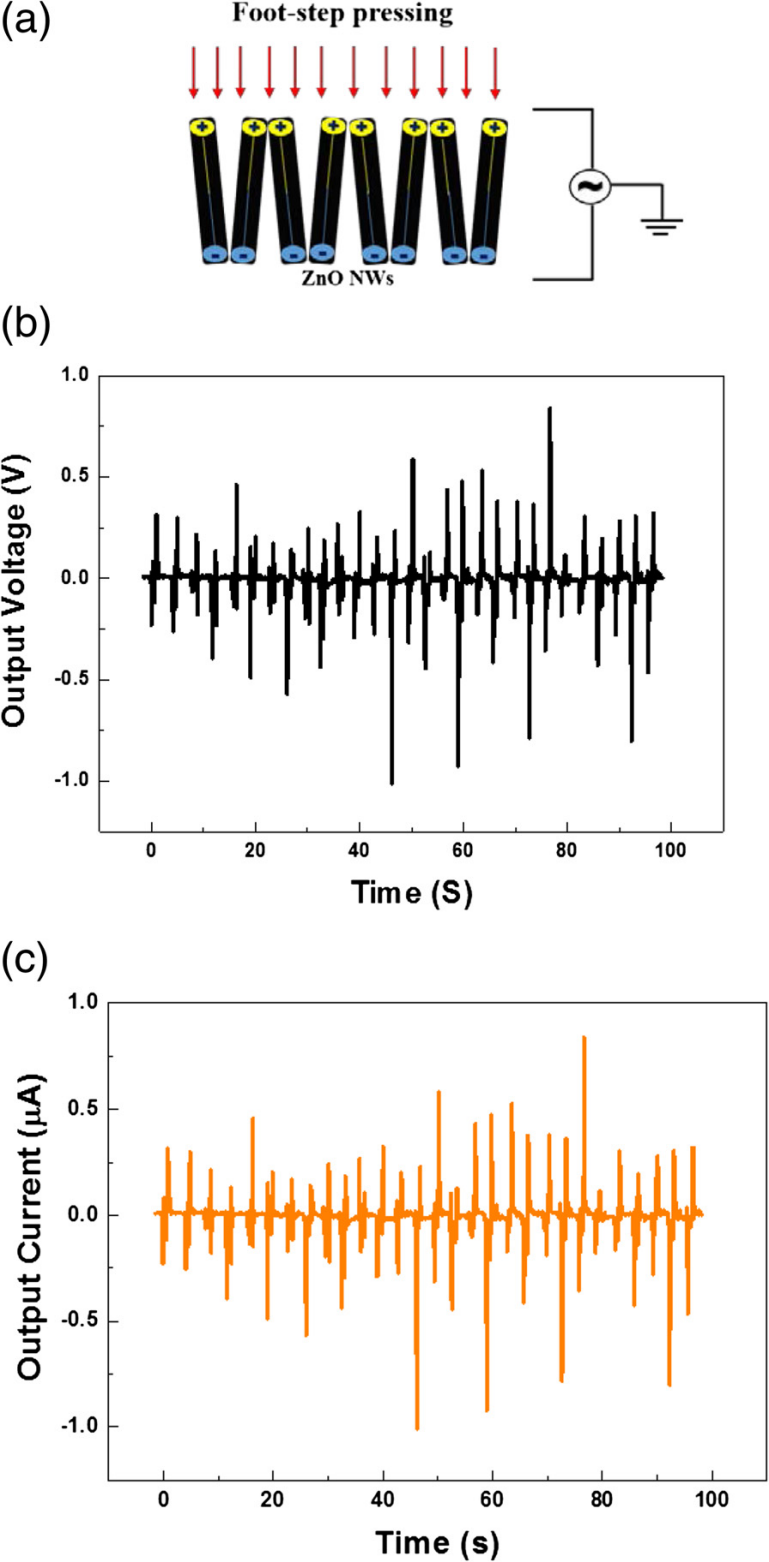
**a** Schematic diagram of the piezoelectric voltage generation under footstep pressing. **b**, **c** The output voltage and current as a function of time under repeated footsteps

The working principle of the NG is related to the coupling of the piezoelectric and the semiconducting properties. When a stress is applied by an external force, the ZnO NWs grown parallel to the *c*-axis are under uniaxial compression. A negative and positive piezoelectric potential occur, respectively, at the top and bottom sides of the ZnO NWs; the corresponding transient current flows from the top to the bottom through the external circuit, which is then detected as an electric pulse. As the compressive strain is released by the removal of the external force, the piezoelectric potential in the NWs disappears. As a result, the electrons flow back via the external circuit, creating an electric pulse in the opposite direction [24]. Figure 2a is a schematic diagram illustrating the mechanism of electric power generation of ZnO NWs under footstep pressing. These electric pulses can be accumulated, and the amount can significantly be enhanced by parallel and/or series connections for many nanogenerators.

A single NG with an area 12 cm^2^ reached an output open circuit voltage and short circuit current of about 1 V and 1 μA, respectively. As shown in Fig. 2b, c) the obtained results are considered as an improvement in the harvested output voltage observed compared to previously reported results from similar configuration, i.e., ZnO NWs/PVDF combination [25]. In [25], the authors used Cr/Au as top and bottom contact, while we have used Ag. It is well known that Cr/Au on ZnO material leads to form a Schottky contact while Ag forms ohmic contact when deposited on ZnO material. The Schottky contact to pass current (in forward bias) usually causes a voltage drop of few hundreds of millivolt. This voltage drop will be reduced from the harvested electrical power of the NG, and hence, a configuration with ohmic contact would be expected to yield a higher value of the harvested electrical energy. In addition, and considering our previous study in [14], we have observed that similar configurations on different substrates (flexible plastic or flexible porous paper substrates) yield different harvested electrical power. An improvement with a factor of 100 was observed when using flexible porous paper instead of flexible plastic [14]. Hence, the main reasons proposed for the observed improvement are the type of contact and the substrate used.

**Fig. 3:**
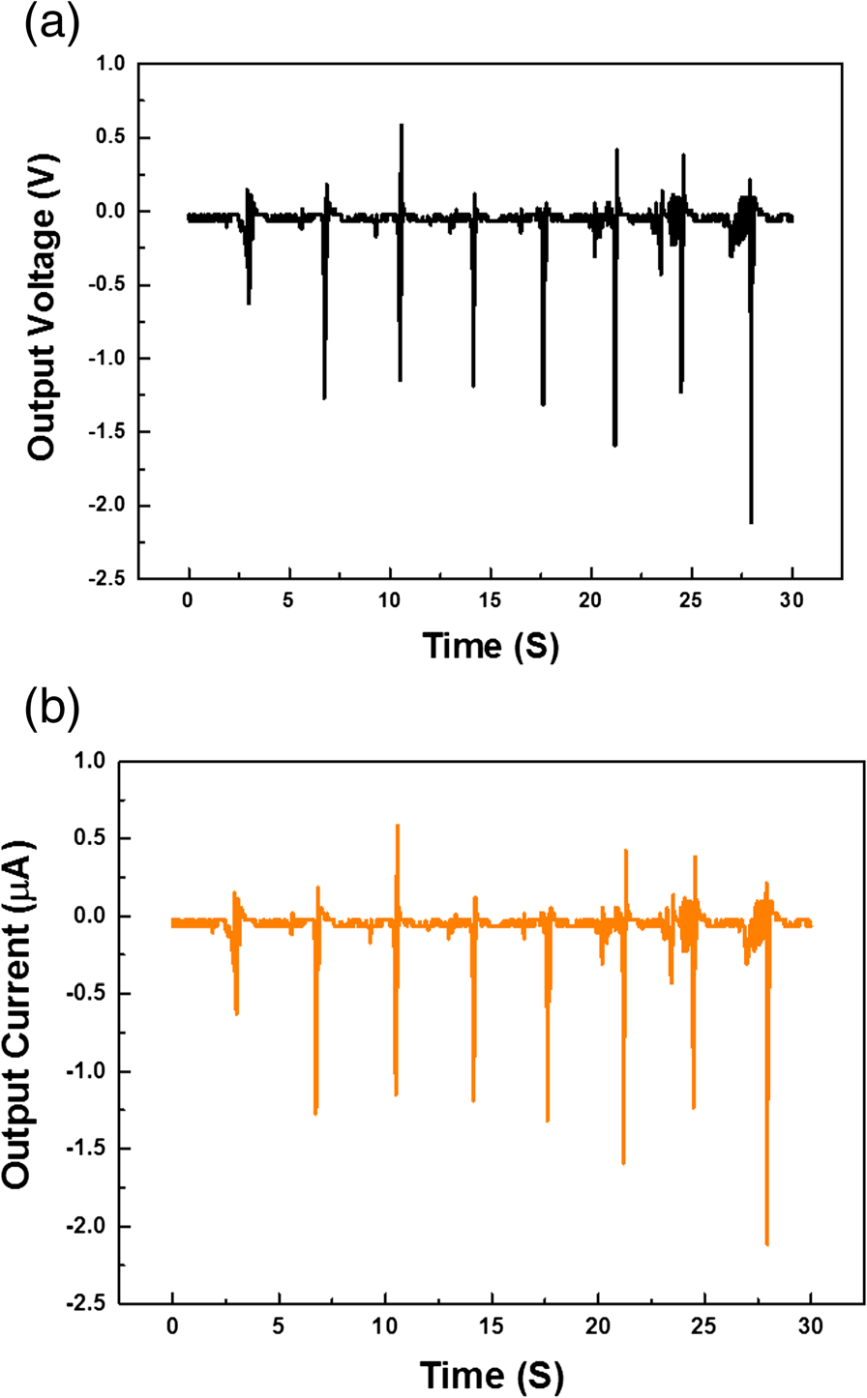
The output voltage and current (from two NGs connected in series) as a function of time under repeated footsteps

Serial connection of many NGs is an effective method for increasing the output voltage. Figure 3a, b show the output signal from a two-layer-structured NG integrated device. The open circuit voltage and short circuit current reach up to a maximum of 2.0 V and 2 μA, respectively.

**Fig. 4:**
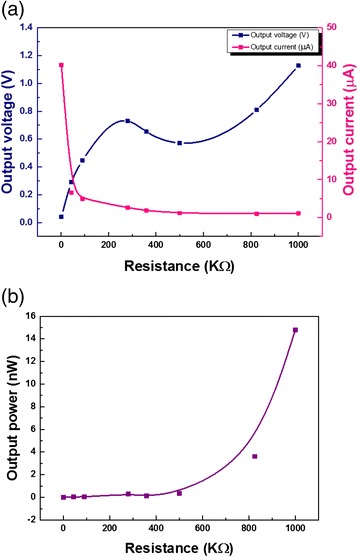
The average output **a** voltage and current and **b** power as a function of the resistance under one footstep cycle

The typical output characteristics of the NG were systematically studied by periodically pressing and releasing at a controlled frequency and amplitude. Figure 4a illustrates the output performance of the NG under different external loads (measured with Agilent DSO-X-2012A). As we can see, the output peak voltage is increased with external load resistance, from 0.04 V at 1 KΩ to 1.13 V at 1 MΩ. Figure 4b shows the output signal power above the noise floor as a function of load resistance. The signal power is estimated using Eq. (1):

1 $$ P = \frac{{\left({V}_{\mathrm{rms}}-{V}_{\mathrm{noise}}\right)}^2}{R} $$

where *P* is the power of the generated signal above the noise floor, V_rms_ is the root mean square (RMS) voltage, V_noise_ is the RMS noise, and *R* is the resistance.

### Wireless Properties

In order to transmit a signal from the NG through wireless scenario, a wireless sensor network (WSN) developed at Linköping University (Sweden) was used [26–28]. The WSN is based on a ZigBee specification. The WSN consists of gateway, routers, and end devices. The gateway and routers are the mains-powered components and the end devices for sensing are battery-powered.

**Fig. 5:**
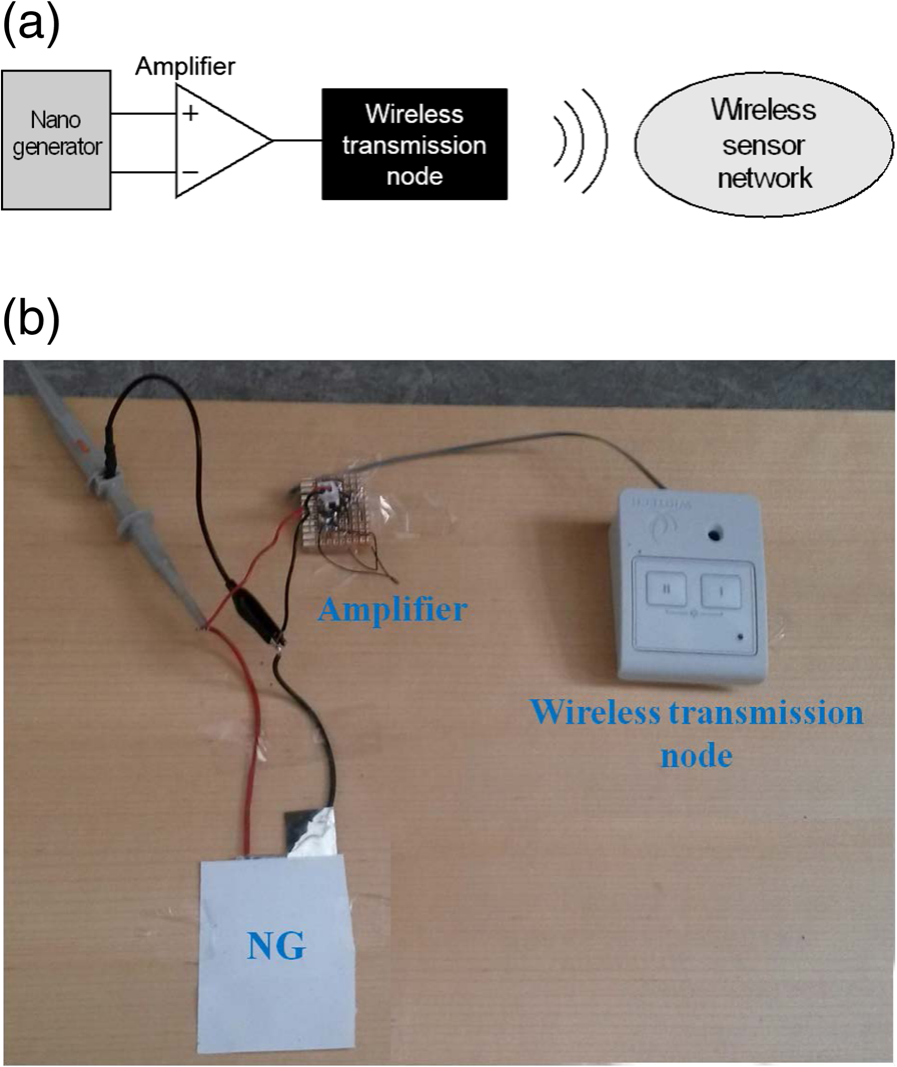
Complete electronic circuit during measurements. **a** Schematic and **b** photograph

**Fig. 6:**
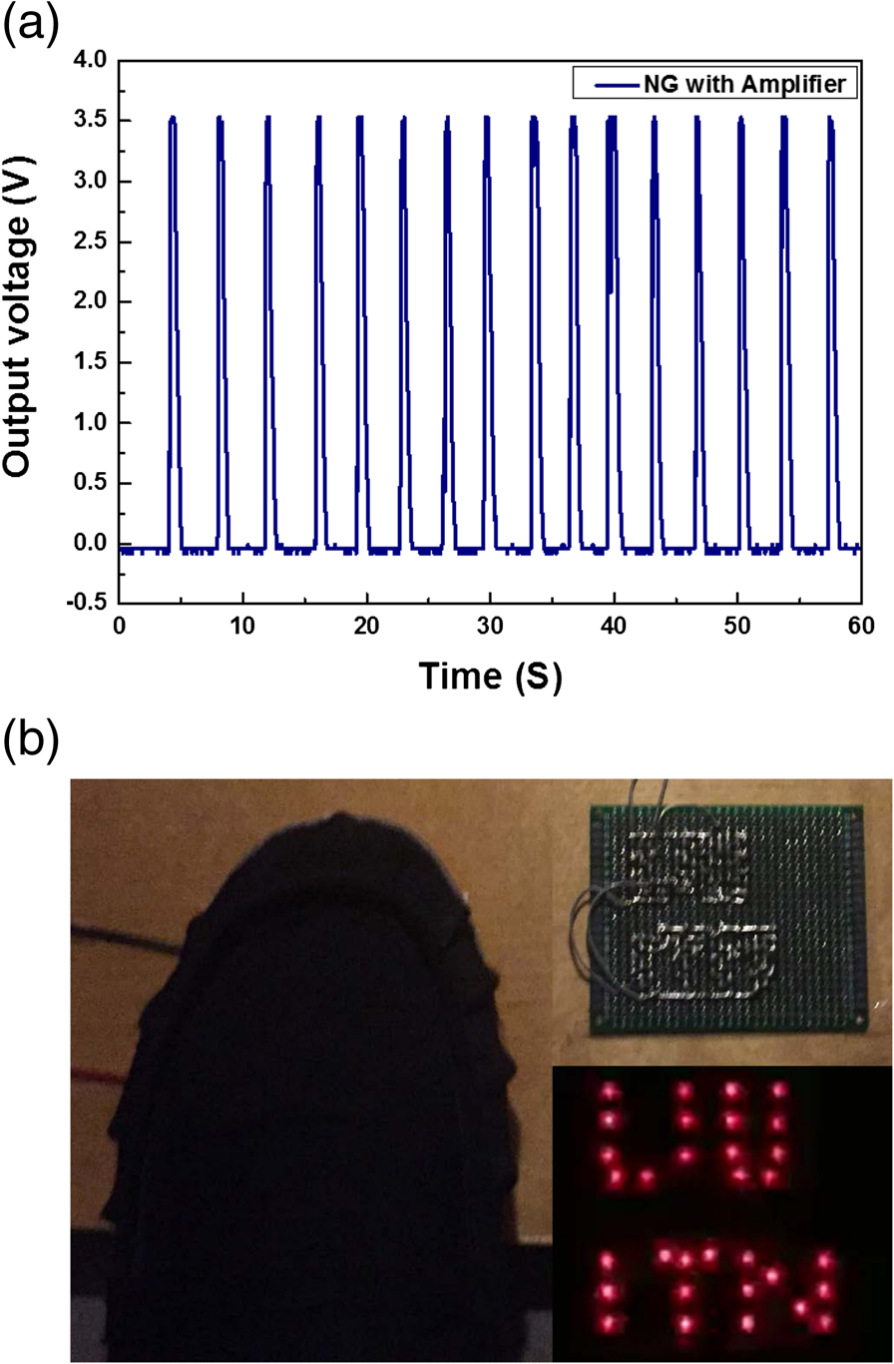
**a** The average output voltage as a function of time under one footstep when the amplifier is connected to one NG. **b** Digital photograph of the prototype of the energy-harvesting circuits connected to LED display triggered by foot pressing

The piezoelectric NG is connected to the end device through an amplifier as shown in Fig. 5a, b). The end device (wireless transmission node) sends a message, when an event occurs. The message is received by the gateway between the wireless sensor network and the Internet and saved in the cloud. An interrupt should be triggered for the event to occur. It is triggered by the rising edge of the digital signal. The digital signal is considered to be logically 0 when the voltage is lower than 0.5 V and digital 1 when the voltage is higher than 2.5 V. As the maximum voltage of a single NG is less than 2.5 V, an amplifier is used to ensure stable operation. An operational amplifier in non-inverting configuration is used to amplify the signal from a single NG by 40 times Fig. 6a. It should be noted that the amplifier is powered by a DC battery, while the ZnO NWs/PVDF-TrFE NG is the only self-powered component in the circuit shown in Fig. 5a. The amplifier consumes 0.6 μA in idle mode, which makes it suitable for battery-powered applications. The input resistance of the amplifier is 3.3 GΩ. Figure 6b shows the captured image of a commercial red LED integrated into the circuit shown in Fig. 5. Upon walking on the ZnO NWs/PVDF-TrFE NG, the LEDs turn to the on state and were lighting up. The commercial microelectronic LEDs circuit has successfully been operated/triggered by piezoelectric energy from a single NG device. Due to the unstable output voltage form our present NG, we used a battery-driven amplifier. Then, by applying one footstep, we were able to detect a signal appearing as a single peak. Another innovative self-powered system with wireless data transmission using ZnO nanowires with electrical energy storage component has been previously published [29]. In this work, the wireless system was battery free and the configuration of the NG was fabricated on flexible polyester of thickness around 220 μm. Then, five layers of ZnO nanowires were placed on each other to harvest mechanical energy. By applying three cycles to this five-layer configuration, the stored electrical power was enough to transmit the signal.

## Conclusions

Utilizing renewable and sustainable power sources is indispensable for the development of green electronics and can be of potential to wireless sensor networks. In this work, we design and fabricate a paper-based nanogenerator (NG) utilizing piezoelectric ZnO nanowires grown hydrothermally on a paper substrate. The fabricated NG is capable of harvesting ambient mechanical energy from various kinds of human motions, such as footsteps. The generated electric output from a single ZnO NWs/PVDF-TrFE NG has been used to serve as low-frequency self-powered triggering sensor. Using the demonstrated piezoelectric footstep sensor, a wireless transmission was operated successfully.
